# Exendin-4, a glucagon-like peptide-1 receptor agonist, inhibits hyperglycemia-induced apoptosis in myocytes by suppressing receptor for advanced glycation end products expression

**DOI:** 10.3892/etm.2014.1873

**Published:** 2014-07-31

**Authors:** BO YI, XIAORONG HU, ZHONGYUAN WEN, TING ZHANG, YULI CAI

**Affiliations:** 1 Department of Endocrinology, Renmin Hospital of Wuhan University, Wuhan, Hubei 430060, P.R. China; 2 Department of Cardiology, Renmin Hospital of Wuhan University, Wuhan, Hubei 430060, P.R. China

**Keywords:** glucagon-like peptide-1, receptor for advanced glycation end products, hyperglycemia, apoptosis, cardioprotection

## Abstract

Activation of the receptor for advanced glycation end products (RAGE) axis may have an important role in apoptosis. Glucagon-like peptide-1 (GLP-1) is a gut hormone that has been proposed as a therapeutic target for the treatment of diabetes, and GLP-1 receptor agonists have been reported to protect against myocardial injury associated with diabetes. The aim of the present study was to investigate the cardioprotective mechanism of exendin-4 (EX-4), a GLP-1 receptor agonist, against myocardial cell apoptosis induced by hyperglycemia. Neonatal rat ventricular myocytes were prepared by enzymatic dissociation and then cultured with high levels of glucose (HG) in the presence or absence of EX-4. Cell apoptosis was detected using an annexin V-fluorescein isothiocyanate/propidium iodide kit, and cell viability was measured using an MTT assay. RAGE expression levels and the activity of caspase-3 were assessed by western blot analysis. The results demonstrated that the incubation of myocytes with HG led to a time-dependent activation of RAGE, and the protein expression of RAGE was increased at 6 h and peaked at 24 h (P<0.05). Hyperglycemia was also found to significantly decrease cell viability and increase apoptosis (P<0.05). In addition, EX-4 significantly inhibited hyperglycemia-induced RAGE expression and the apoptosis of myocytes, and improved cell viability in a dose-dependent manner (P<0.05). When the concentration of EX-4 was 10 nM, the myocardial cell viability was significantly improved, and the levels of RAGE expression and apoptosis were significantly decreased compared with those in the HG group in the absence of EX-4 (P<0.05). Therefore, the results from the present study suggest that the cardioprotective effect induced by EX-4, a GLP-1 receptor agonist, against diabetic cardiomyopathy may be associated with the inhibition of RAGE expression.

## Introduction

Diabetes causes various cardiovascular complications, for example diabetic cardiomyopathy, which has become the major cause of morbidity and mortality among patients with diabetes. Previous studies have suggested that cardiomyocyte apoptosis has a key role in diabetic cardiac damage in animals and humans (1–3). Hyperglycemia is known to cause apoptosis in cardiomyocytes, which leads to diabetic cardiomyopathy (1,4,5). Attenuation of hyperglycemia-induced cardiomyocyte cell death has been shown to prevent the progression of cardiac complications associated with diabetes (1,6).

The multi-ligand receptor for advanced glycation end products (RAGE), which was first identified as receptor for the advanced glycation end products (AGEs), is a signal transduction receptor belonging to the immunoglobulin superfamily (7). RAGE is expressed on multiple cell types, including vascular cells, inflammatory cells, neurons (central and peripheral nervous systems) and glomerular epithelial cells (8), and hyperglycemia has been shown to directly induce RAGE expression in endothelial cells (9) and retinal Müller glia (10). However, RAGE interacts with ligands other than AGEs; non-AGE ligands of RAGE include high mobility group box 1 (HMGB1), members of the S100/calgranulin family, amyloid-β peptide and Mac-1 (8). Diabetic RAGE-null mice are significantly protected from the adverse effects of ischemia and reperfusion injury of the heart. In addition, important markers of apoptosis, specifically, caspase-3 activity and cytochrome *c* release, have been demonstrated to be decreased in the hearts of diabetic RAGE-null mice compared with those in wild-type diabetic littermates during myocardial ischemia and reperfusion (11). Therefore, this suggests that hyperglycemia-induced RAGE expression may have an important role in diabetic cardiac damage.

Glucagon-like peptide-1 (GLP-1), a gut hormone secreted in a nutrient-dependent manner, stimulates insulin secretion and inhibits glucagon secretion and gastric emptying (12). Therefore, GLP-1 has been proposed to be a potential therapeutic target for the treatment of patients with type 2 diabetes mellitus. Clinical studies have shown that GLP-1 improves endothelial function in patients with type 2 diabetes (13), and transient GLP-1 administration is able to improve cardiovascular outcomes in patients with myocardial infarction (MI) (14) or congestive heart failure (CHF) (15,16). Furthermore, previous studies have suggested that exendin-4 (EX-4), a GLP-1 receptor agonist, may protect against myocardial ischemia and reperfusion injury and reduce rates of oxidative phosphorylation in the adult rat heart (17,18), as well as prevent cardiac remodeling in the hearts of rats with type 1 diabetes (19). However, the mechanism by which EX-4 protects against myocardial injury associated with diabetes remains unclear. Therefore, the present study investigated whether EX-4 inhibits hyperglycemia-induced apoptosis in myocardial cells by suppressing RAGE expression.

## Materials and methods

### Cell culture and treatment

Neonatal rat ventricular myocytes were prepared from the hearts of Sprague-Dawley rats (aged between 1 and 3 days) by enzymatic dissociation, as previously described (20). Briefly, the rats were euthanized and their hearts excised. Following homogenization using a scalpel, the heart tissue was treated with 0.1% (w/v) collagenase for 20 min at 37°C, and then incubated with 0.25% (w/v) trypsin overnight at 4°C. Experimental protocols were conformed to the Guide for the Care and Use of Laboratory Animals published by the National Institutes of Health, and were approved by Wuhan University (Wuhan, China) Cardiomyocytes were enriched by Percoll gradient centrifugation (Amersham Pharmacia Biotech, Piscataway, NJ, USA) and plated at a density of 5×10^5^/ml in Dulbecco’s modified Eagle medium supplemented with 15% (v/v) fetal calf serum at 37°C and 5% (v/v) CO_2_. Following incubation in serum for 24 h, the cells were washed and cultured in serum-free medium for 24 h, and the cultures were then subjected to different treatments.

To determine the effect of glucose on the expression of RAGE, cells were exposed to high levels of glucose (HG; Sigma-Aldrich, St. Louis, MO, USA) for different time periods (0, 6, 12, 24 and 48 h). A total of 25 mmol/l D-glucose was used for the HG treatments, compared with 5 mmol/l D-glucose used as the normal control (NG), as previously described (4,5). To exclude a hyperosmolar effect, the same concentration of mannitol (25 mmol/l; Sigma-Aldrich) was used in control cultures. For the purpose of testing the cardioprotective effect of GLP-1, cells were treated with 25 mmol/l D-glucose either with or without EX-4 (Sigma-Aldrich). The concentrations of EX-4 tested were 0.1, 1 and 10 nM.

### 3-(4,5-Dimethylthiazolyl-2)-2,5-diphenyltetrazolium bromide (MTT) assay for the determination of cell viability

The cultured cells were seeded at a density of 1×10^5^/ml per well. Subsequently, MTT (Sigma-Aldrich) was added (final concentration, 5 mg/ml) to each well. The cells were incubated for 4 h, and then, following the addition of 100 μl 10% sodium dodecyl sulfate (SDS) and 0.01 N HCl to dissolve the crystals, the cells were incubated for a further 16 h. The absorbance was determined using an automatic microplate reader at a wavelength of 570 nm. The relative cell viability was expressed as a percentage of the cell viability of the control group.

### Annexin V-fluorescein isothiocyanate (FITC)/propidium iodide (PI) staining for detecting cardiac myocyte apoptosis

Apoptosis was detected using an annexin V-FITC/PI kit (BD Pharmingen, San Diego, CA, USA) in accordance with the manufacturer’s instructions, as previously described (20). Briefly, cells were harvested after 24 h and washed with phosphate-buffered saline, prior to being pelleted by centrifugation at 1500 × g for 5 min. Cells were resuspended in 300 μl binding buffer (1×10^5^ cells/ml) followed by staining with 5 μl annexin V-FITC and 5 μl PI for 15 min at room temperature in the dark. The percentage of cell apoptosis was then determined using flow cytometry with a BD FACSCalibur platform (BD Biosciences, Franklin Lakes, NJ, USA).

### Western blot analysis

Cardiomyocytes were lysed using a Protein Complete Lysis kit (Beyotime Institute of Biotechnology, Haimen, China). The protein concentration in each sample was determined using a Protein Assay kit (Beyotime Institute of Biotechnology) using bovine serum albumin as a standard. For the immunoblot analysis, proteins were separated using SDS-PAGE, and then transferred onto a polyvinylidene fluoride (PVDF) membrane as previously described (5,20). The membranes were probed using antibodies against RAGE and caspase-3 (Cell Signaling Technology, Inc., Danvers, MA, USA), followed by horseradish peroxidase-conjugated secondary antibodies (Santa Cruz Biotechnology, Inc., Santa Cruz, CA, USA). The membranes were visualized using an enhanced chemiluminescence system (Beyotime Institute of Biotechnology). The levels of protein expression were normalized against glyceraldehyde-3-phosphate dehydrogenase (GAPDH) expression.

### Statistical analysis

Statistical analysis was performed using SPSS software, version 13.0 (SPSS, Inc., Chicago, IL, USA). All values are expressed as the mean ± standard deviation. The Student’s t-test was used for between-group comparisons. Welch’s analysis of variance was used for comparisons among groups, and the Student-Neuman-Keuls or Dunnett’s T3 test was used for post-hoc multiple comparisons. P<0.05 was considered to indicate a statistically significant difference.

## Results

### Effect of HG on RAGE expression

**Figure 1:**
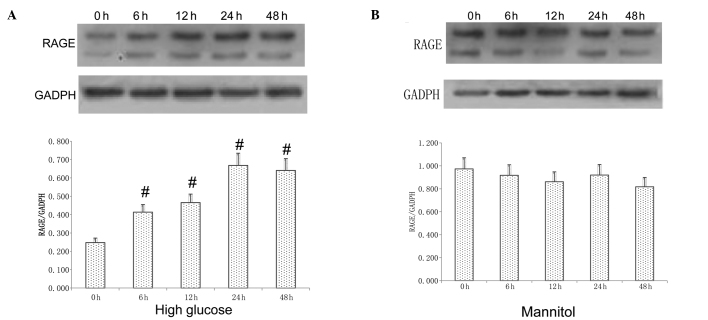
Effect of high glucose on RAGE expression. (A) Incubation with high glucose (25 mmol/l) for 0, 6, 12, 24 and 48 h. (B) Incubation with 25 mmol/l mannitol for 0, 6, 12, 24 and 48 h. Data are presented at the mean ± standard deviation (n=3). ^#^P<0.05, vs. 0 h. RAGE, receptor for advanced glycation end products.

Incubation of myocytes with HG led to a time-dependent activation of RAGE expression compared with that observed in a low-glucose environment, and the protein expression of RAGE was increased at 6 h and peaked at 24 h (P<0.05; Fig. 1A). However, the protein levels of RAGE in myocytes were not changed following treatment with 25 mmol/l mannitol (Fig. 1B).

### Cell viability

**Figure 2:**
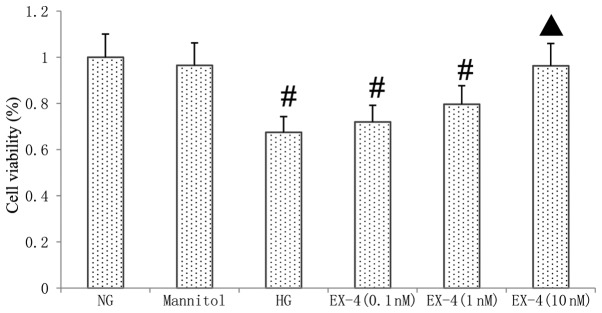
Effect of HG and EX-4 on cell viability. Cardiomyocytes were treated with HG or mannitol with/without EX-4, a GLP-1 receptor agonist. After 24 h, cell viability was analyzed using the MTT assay. ^#^P<0.05 vs. NG group, ^▲^P<0.05 vs. HG group. NG, 5 mmol/l glucose; mannitol, 25 mmol/l mannitol; HG, high glucose concentration of 25 mmol/l; EX-4 (0.1 nM), 0.1 nM EX-4 and 25 mmol/l glucose; EX-4 (1 nM), 1 nM EX-4 and 25 mmol/l glucose; EX-4 (10 nM), 10 nM EX-4 and 25 mmol/l glucose. NG, normal glucose; EX-4, exendin-4; HG, high glucose; MTT, 3-(4,5-dimethylthiazolyl-2)-2,5-diphenyltetrazolium bromide.

As shown in Fig. 2, the treatment of myocytes with HG for 24 h resulted in significantly decreased cell viability compared with that in the NG group (P<0.05), as shown by an MTT assay. In the myocardial cells cultured with HG and various concentrations of EX-4, the cell viability was increased by EX-4 in a dose-dependent manner. At a concentration of 10 nM EX-4, the cell viability of the myocardial cells was significantly improved compared with that in the HG group (P<0.05).

### Effect of EX-4 on HG-induced myocyte apoptosis

**Figure 3:**
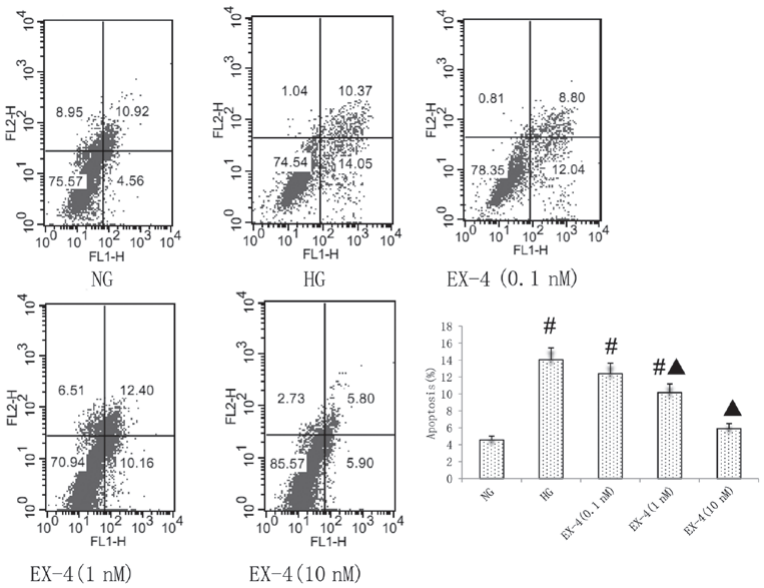
Effect of EX-4 on glucose-induced myocyte apoptosis. ^#^P<0.05, vs. the NG group, ^▲^P<0.05, vs. the HG group. NG, normal glucose; EX-4, exendin-4; HG, high glucose.

According to the results from the MTT assay, the effects of different concentrations of EX-4 (0.1–10 nM) on myocardial cell apoptosis were determined. Flow cytometric analysis demonstrated that EX-4 inhibited the apoptosis of neonatal myocytes induced by HG in a dose-dependent manner (P<0.05; Fig. 3).

**Figure 4:**
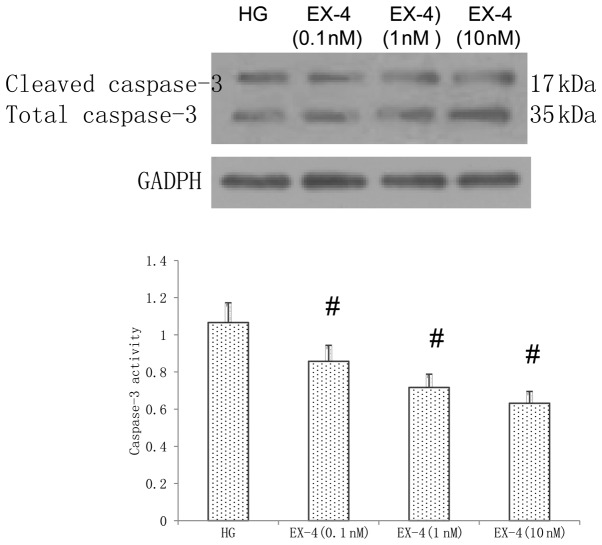
Effect of EX-4 on the HG-induced activation of caspase-3 in cardiomyocytes. ^#^P<0.05, vs. the HG group. EX-4, exendin-4; HG, high glucose; GADPH, glyceraldehyde-3-phosphate dehydrogenase.

Cleaved caspase-3 is a key executor in the apoptotic process, which is induced by HG. Therefore, in the present study, the expression of caspase-3 was detected using western blot analysis. As shown in Fig. 4, the HG-induced activity of caspase-3 was inhibited in the cells treated with EX-4 in a dose-dependent manner compared with that in the cells in the HG group (P<0.05).

### Effect of EX-4 on RAGE expression

**Figure 5:**
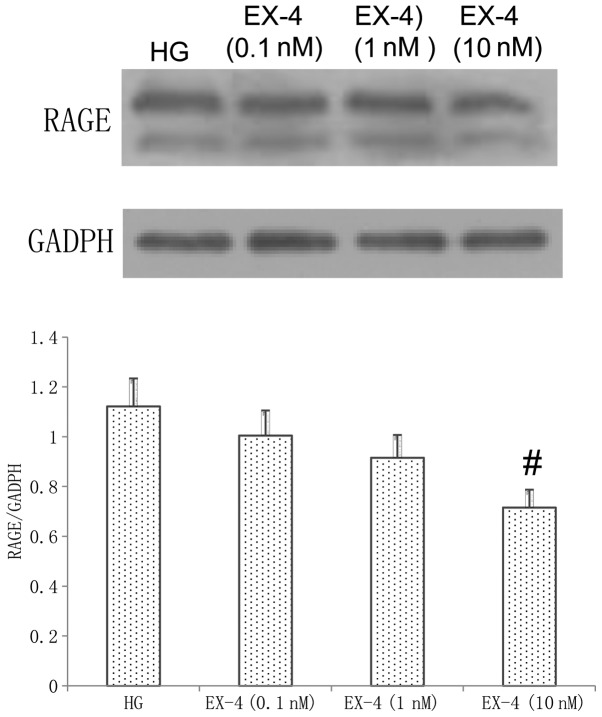
Effect of EX-4 on HG-induced RAGE expression. ^#^P<0.05, vs. the HG group. EX-4, exendin-4; HG, high glucose; RAGE, receptor for advanced glycation end products; GADPH, glyceraldehyde-3-phosphate dehydrogenase.

As shown in Fig. 5, following incubation with HG and EX-4 for 24 h, EX-4 inhibited the RAGE expression induced by HG. At a concentration of 10 nM EX-4, the expression of RAGE was significantly improved compared with that in the HG group (P<0.05).

## Discussion

In the present study, it was demonstrated that hyperglycemia increases the expression of RAGE and induces apoptosis in myocytes. In addition, EX-4, a GLP-1 receptor agonist, was shown to protect against myocardial apoptosis in a dose-dependent manner. The inhibitory effect of EX-4 on apoptosis may be via a reduction in caspase-3 activity and the inhibition of RAGE expression. These results indicate that the cardioprotective effect induced by EX-4 during diabetic cardiomyopathy may be associated with the inhibition of RAGE expression.

Hyperglycemia, which directly causes abnormalities at the cardiac myocyte level, including apoptosis, is the main pathogenetic factor of diabetic cardiomyopathy. Anderson *et al* (21) demonstrated that the myocardium in patients with diabetes has a greater overall propensity for mitochondrial-dependent cell death, possibly as a result of metabolic stress-imposed changes that have occurred within the mitochondria. In addition, a previous study demonstrated that hyperglycemia directly induces apoptosis in the myocardium, and it is mediated by activation of the cytochrome *c*-activated caspase-3 pathway (1). The results from the present study are in accordance with these observations.

Furthermore, in a previous study we demonstrated that HMGB1 promotes the apoptosis of neonatal myocytes in a dose-dependent manner (20). Since HMGB1 is a ligand of RAGE, the role of the RAGE axis in hyperglycemia-induced apoptosis in myocytes was investigated in the present study. The results demonstrated that hyperglycemia increases RAGE expression in myocardial cells, and the protein expression of RAGE was increased at 6 h and peaked at 24 h. Yao and Brownlee (9) previously found that HG increase RAGE and HMGB1 expression, and that this effect is mediated by reactive oxygen species-induced methylglyoxal, the major substrate of glyoxalase 1. Furthermore, the interaction between HMGB1 and RAGE has been shown to be important in neuronal cell apoptosis (22), and the RAGE axis is also involved in the apoptosis of a number of different cell types, including pancreatic β cells (23), esophageal squamous cell carcinoma (24) and neuronal cells (22). In addition, RAGE-null mice with diabetes have been previously demonstrated to be significantly protected against the adverse impact of ischemia/reperfusion (I/R) injury of the heart, and key markers of apoptosis, including caspase-3 activity and cytochrome *c* release, were found to be decreased in the hearts of RAGE-null mice with diabetes compared with those in wild-type mice with diabetes during myocardial I/R (11). In combination, these results suggest that hyperglycemia-induced RAGE expression has an important role in diabetic cardiac damage and the RAGE axis may be a therapeutic target for diabetic cardiovascular complications.

As a novel hypoglycemic agent, GLP-1 acts through a distinct heptahelical G-protein-coupled receptor (GLP-1R). Since this receptor is abundantly expressed in β cells and throughout the gut, heart, vascular smooth muscle cells, endothelial cells, kidney, lung and peripheral nervous system (25), GLP-1 appears to modulate a wide variety of physiologic effects. The cardioprotective effect induced by GLP-1 and GLP-1 receptor agonists has been demonstrated in previous studies (13–19); however, the mechanism has yet to be elucidated. Ravassa *et al* (26) demonstrated that GLP-1 (100 nM) inhibits staurosporine-induced apoptosis in murine HL-1 cardiomyocytes, and GLP-1 also inhibits palmitate- and ceramide-induced DNA fragmentation, which is an integral part of apoptosis. However, Chen *et al* (27) demonstrated that treatment with EX-4 had no effect on LPS-induced apoptosis in H9c2 cardiomyoblast cells. The reasons for the different results may be due to the different cell types, the different GLP-1 doses used and the different apoptosis models. In the present study the effects of various EX-4 doses (0.1, 1 and 10 nM) on hyperglycemia-induced apoptosis in neonatal rat ventricular myocytes were investigated. The results indicated that EX-4 protects against myocardial hyperglycemia-induced apoptosis in a dose-dependent manner via a downregulation of caspase-3 cleavage; this effect was most significant at a 10-nM concentration of EX-4.

Hyperglycemia may activate the RAGE axis, which contributes to myocyte apoptosis; therefore, in the present study, it was investigated whether EX-4 was able to suppress RAGE expression in myocardial cells. The results showed that the expression of RAGE in myocytes was significantly decreased by EX-4, and this inhibitory effect was greatest in the cells treated with 10 nM EX-4. Ishibashi *et al* (28,29) previously demonstrated that GLP-1 may directly act on mesangial cells and human umbilical vein endothelial cells via GLP-1R and it may work as an anti-inflammatory agent against AGEs by reducing RAGE expression. The results from the present study also indicated that EX-4 is able to inhibit the protein expression of RAGE that is induced by hyperglycemia in myocytes.

In conclusion, the results from the present study suggest that the cardioprotective effect induced by a GLP-1 receptor agonist (EX-4) during diabetic cardiomyopathy may be associated with the inhibition of RAGE expression. However, there were certain limitations in the present study, as the effects of EX-4 on RAGE expression and apoptosis were only observed following their induction by hyperglycemia. Therefore, the precise mechanisms underlying the observations require further investigation.
